# Spatial transcriptomics in glioblastoma: is knowing the right zip code the key to the next therapeutic breakthrough?

**DOI:** 10.3389/fonc.2023.1266397

**Published:** 2023-10-17

**Authors:** Jack M. Shireman, Lingxin Cheng, Amiti Goel, Diogo Moniz Garcia, Sanil Partha, Alfredo Quiñones-Hinojosa, Christina Kendziorski, Mahua Dey

**Affiliations:** ^1^Department of Neurosurgery, University of Wisconsin School of Medicine and Public Health, University of Wisconsin-Madison (UW) Carbone Cancer Center, Madison, WI, United States; ^2^Department of Biostatistics and Medical Informatics, University of Wisconsin School of Medicine and Public Health, Madison, WI, United States; ^3^Department of Neurosurgery, Mayo Clinic, Jacksonville, FL, United States

**Keywords:** glioblasoma, spatial transcriptomics, bioinformatics, cancer, personalized medicine

## Abstract

Spatial transcriptomics, the technology of visualizing cellular gene expression landscape in a cells native tissue location, has emerged as a powerful tool that allows us to address scientific questions that were elusive just a few years ago. This technological advance is a decisive jump in the technological evolution that is revolutionizing studies of tissue structure and function in health and disease through the introduction of an entirely new dimension of data, spatial context. Perhaps the organ within the body that relies most on spatial organization is the brain. The central nervous system’s complex microenvironmental and spatial architecture is tightly regulated during development, is maintained in health, and is detrimental when disturbed by pathologies. This inherent spatial complexity of the central nervous system makes it an exciting organ to study using spatial transcriptomics for pathologies primarily affecting the brain, of which Glioblastoma is one of the worst. Glioblastoma is a hyper-aggressive, incurable, neoplasm and has been hypothesized to not only integrate into the spatial architecture of the surrounding brain, but also possess an architecture of its own that might be actively remodeling the surrounding brain. In this review we will examine the current landscape of spatial transcriptomics in glioblastoma, outline novel findings emerging from the rising use of spatial transcriptomics, and discuss future directions and ultimate clinical/translational avenues.

## Introduction

Developmental patterning, the process of determination of final fate and ultimate locations for all cells originating from the initial cluster of stem cells in a developing embryo, is essential for normal development of a multicellular organism. A chief driving factor behind developmental patterning is cellular location and exposure to gradient signaling cascades such as SHH and WNT pathway signaling (1–6). Further developmental complexity is setup within nearly all organ systems throughout the body relying on interactions between cellular neighbors (7–9). During early embryonal development, brain undergoes complex developmental patterning for separation of central nervous system (CNS) and peripheral nervous system (PNS) as well as the development and migration of cells throughout the cerebral cortex ranging from axon guidance to synapse formation (4, 10–13). Firmly rooted in this developmental complexity is cellular location, which until recently was almost entirely missing from the field of large-scale genomic sequencing.

Understanding cellular behavior in its native location and organ system is fundamental to not only basic developmental biology but also to defining a pathological condition and developing relevant therapeutics. Since the development of mRNA analysis techniques, our understanding of diseases such as cancers have evolved exponentially. By analyzing the overall gene expression profile of the cancer cells, it was not only possible to better define the disease and its behavior, but it has also led to development of several targeted therapies that greatly increased the chance of survival for cancer patients (14, 15). However, as our ability to experimentally probe gene expression advanced from microarray to bulk sequencing and finally to single cell sequencing, and our experimental models and questions became more and more complex, one constant has been missing, location.

Experimentally, the most reliable forms of gene expression analysis have relied on tissue dissociation and even with the advent of single cell sequencing, barcoding for cells has only been able to track cell types, not their original position within their tissue of residence. Dissociation of tissue, especially with complex spatial organization such as the brain, induces cellular stress and exposes cells to foreign environments which impacts the overall transcriptional profile and ultimate results of the experiments (16–18). Even with careful experimental protocols and controls as well as “gentile” dissociation techniques, removing a cell or tissue from its spatial architecture surely loses valuable information on function or disease state.

A pathological condition where the tissue microenvironment plays a curial role in disease progression and therapeutic resistance is glioblastoma (GBM). GBM is the most common primary brain cancer in adults, with limited prognosis and strong resistance to all current therapies (19–21). Multiple factors including intra-tumoral and intra-patient heterogeneity (22–26), systemic immune suppression (26–29), and limited chemotherapeutic access because of the blood brain barrier (30, 31) (BBB) all combine to render GBM as one of the most aggressive and deadly cancers. One of the hypothesized reasons for the aggressiveness and treatment resistance displayed by GBM is its complex spatial organization, seemingly seeking to mimic the spatial architecture of its surrounding microenvironment. The GBM tumor microenvironment (TME) is comprised of endothelial cells, neurons, astrocytes, oligodendrocytes, microglia, tumor-associated macrophages, tumor-infiltrating lymphocytes, and noncellular components such as apocrine and paracrine signaling molecules, exosomes, extracellular matrix (ECM) components, and secreted ECM remodeling enzymes (32, 33). Each of these components play not only individual roles but also orchestrate an incredibly complex and spatially distinct TME in which GBM can evade immune system detection and uncontrollably proliferate.

In recent years, single cell and bulk RNA sequencing, as well as fluorescence *in-situ* hybridization (FISH) have been utilized to study microenvironmental heterogeneity and conduct crude spatial profiling (through location specific biopsy sampling throughout tumors (34)) within the GBM field. This has illustrated the vast intra and inter tumor heterogeneity that is now associated with GBM (35–40). The Ivy Gap project conducted the most comprehensive “spatial sequencing” experiment before the advent of spatial transcriptomics through bulk sequencing and pathological study (*in-situ* hybridization) of biopsy resections from the leading edge, infiltrating tumor, cellular tumor, pseudopalisading cells, and microvascular proliferation (34). The data was released online publicly in 2018 along with a web browser for interactive gene searching throughout the dataset and has amassed nearly 400 citations. Prior to the Ivy Gap work, Sottoriva and colleagues developed a multisampling scheme for 11 GBM patients which was accomplished using Fluorescence Guided Multi Sampling (FGMS) (41). Across the specimens copy number alterations as well as clonal lineage tracking was conducted revealing distinct subclones and unique tumor regions present throughout patients (41). Although transformative for their time, all the above technologies fail to meet the true definition of a spatial transcriptomics experiment conducted within a maintained cellular or tissue environment. This is critical because dissociation of GBM into single cell suspension often requires special techniques as well as enzymes that can alter cell behavior *ex vivo* (16, 17, 42). The newest tissue histology based technological development in the spatial sequencing field aims to address the limitations of previous research tools and allows for spatial visualization of gene expression at near single cell levels, while still providing thousands of capture points for cells affording critical data density (43).

## Spatial transcriptomics

A spatial transcriptomics (ST) experiment starts from a tissue section frozen and sliced to preserve the tissue spatial architecture. While many techniques are available for profiling gene expression spatially across a tissue, the most widely used are the commercial platforms Visium (from 10x Genomics) and GeoMx (from Nanostring) (43). Each of these platforms is compatible with frozen or formalin-fixed paraffin-embedded (FFPE) tissue. In a Visium ST experiment, a tissue sample is sectioned and placed onto a slide containing 4992 spots, with each spot containing millions of capture oligonucleotides with spatial barcodes unique to that spot. The tissue is imaged, typically via Hematoxylin and Eosin (H&E) staining. Following imaging, the tissue is permeabilized to release mRNA which then binds to the capture oligonucleotides, generating a cDNA library consisting of transcripts bound by barcodes that preserve spatial information. Data from a 10x spatial transcriptomics experiment consists of the tissue image coupled with RNA-sequencing data collected from each capture spot.

**Figure 1:**
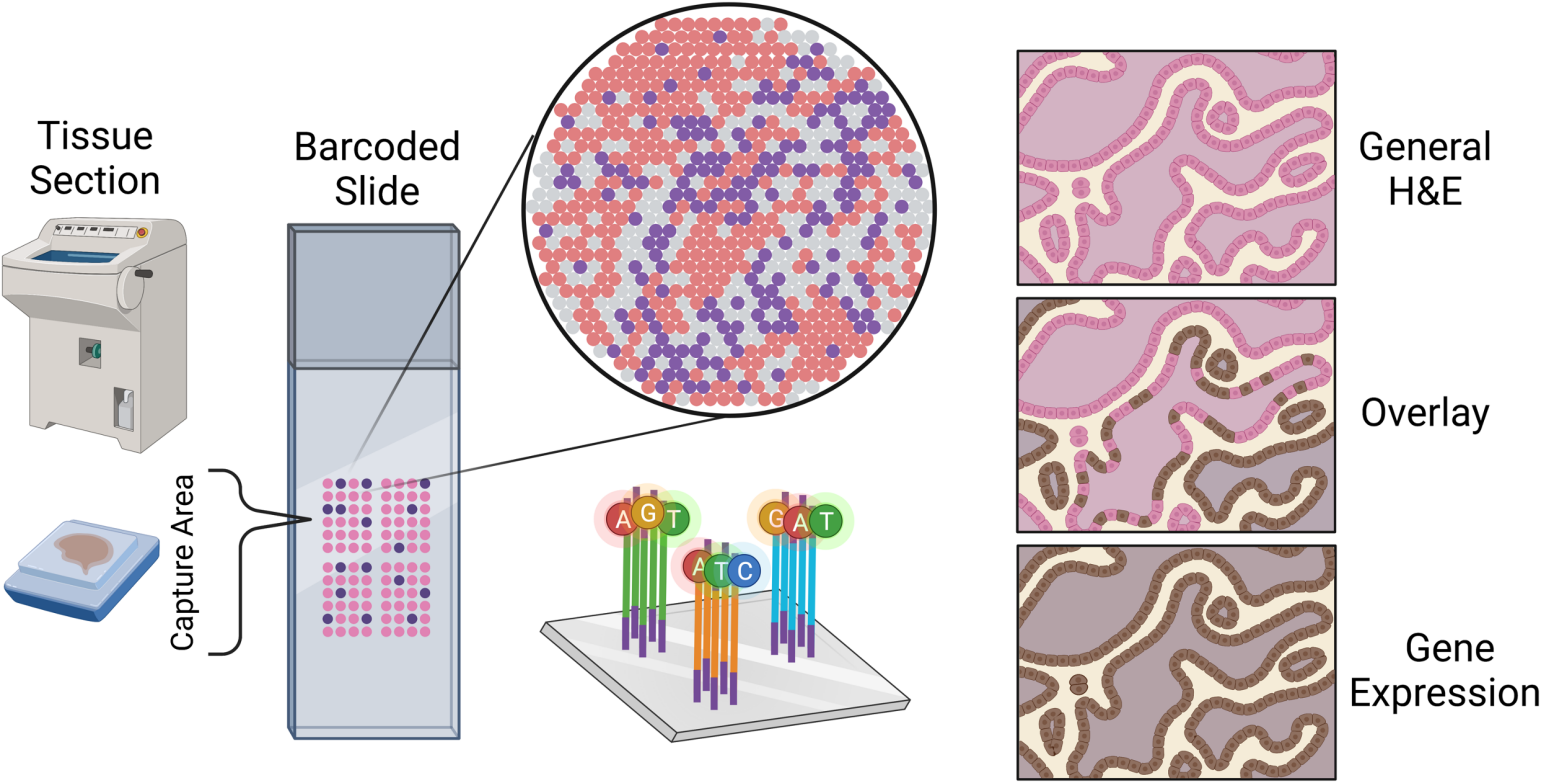
Sketch representation of the slide mounting process of spatial sequencing along with a simulated representation of the data output of a spatial transcriptomics experiment.

Data from a GeoMx experiment is structurally similar in that high-resolution expression data is collected at several locations across a tissue, but typically at lower spatial resolution compared to a Visium experiment. In a GeoMx experiment, a tissue sample is mounted onto a glass slide and incubated with a panel of photocleavable oligonucleotide probes specific to particular mRNAs and/or proteins. High-resolution images of the tissue are obtained in the GeoMx DSP instrument from immunofluorescent staining, colorimetric immunohistochemistry, or *in-situ* hybridization techniques. These images are used for manually selecting regions for expression profiling. These so-called regions of interest (ROIs) are selectively exposed to UV light which cleaves the oligonucleotide tags from the probes or antibodies bound to their targets within the selected regions. The cleaved oligonucleotide tags are then collected and hybridized to a complementary barcode array which is subsequently read by a fluorescence scanner to produce digital counts for each target within the ROIs; alternatively, the cleaved tags can be prepared for sequencing. In either case, the GeoMx technology provides the high-resolution images used to select the ROIs along with count data (or sequenced reads) at each ROI (**Figure 1**).

## Bioinformatic analysis of spatial transcriptomic data

**Figure 2:**
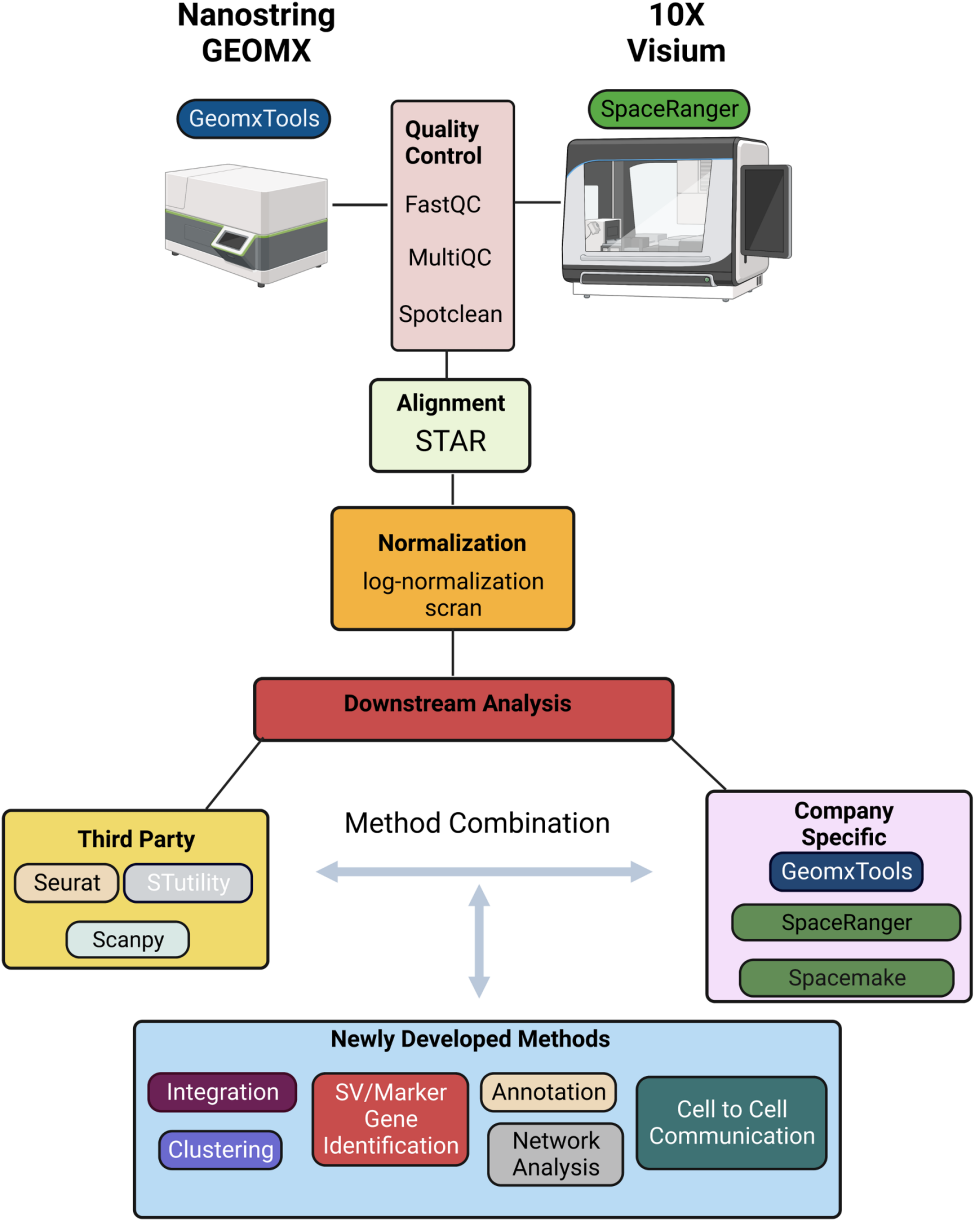
Schematic representation of a typical informatics pipeline used to handle spatial transcriptomic data from either GEOMX or Visium platforms.

There are several software tools and packages available for pre-processing and analysis of ST data including SpaceRanger, GeomxTools, Spacemake, and STutility (10x Genomics Space Ranger 2.0.1, GeomxTools 3.4.0) (44, 45). SpaceRanger is part of the Visium suite of tools while GeomxTools is specific to GeoMx experiments; Spacemake and STutility are applicable to general spatial transcriptomics experiments. Seurat, Scanpy, and other pipelines originally developed for bulk or single-cell RNA-seq analysis have been extended to facilitate ST data pre-processing and analysis. While the details of the pipelines vary, a typical workflow includes alignment of the sequenced reads, quality control, normalization, integration, clustering, and downstream analyses (**Figure 2**). We briefly discuss each of these steps below, first for Visium and then for GeoMx data [detailed reviews of multiple ST technologies and computational methods are provided in (43, 46, 47)].

Sequenced reads can be aligned to a reference transcriptome using any of several alignment tools. Due to its relatively high accuracy, low biases, and speed, STAR is a widely used approach in ST experiments and the default approach implemented is SpaceRanger (10x Genomics Space Ranger 2.0.1). To ensure the accuracy and reliability of the data, it is important to perform quality control following alignment. SpaceRanger provides sequencing and spot-specific metrics to diagnose sequence and spot quality. Given that Visium provides fastq files, most metrics provided in SpaceRanger are the same as those used in fastqc for quality control of bulk or single-cell RNA-seq data. Specifically, percentage of valid barcodes, percentage of valid UMIs, and sequencing saturation metrics are provided and can be used to identify overall sequencing quality; mean reads per spot, median UMI counts per spot, and percentage of genes detected may be used to filter out low-quality spots. The fraction of reads in spots under the tissue is specific to ST experiments and provides a measure of reads with tissue-associated barcodes. Low fraction reads may indicate that many of the reads were not assigned to tissue covered spots due to high levels of ambient RNA, or poor tissue orientation. Finally, spot-swapping is present in most experiments (spot-swapping occurs when reads from a given tissue spot bind probes at a nearby spot) and should be adjusted for using SpotClean (48).

Following alignment and quality control, normalization is often performed to adjust for differences in expression levels that result from technical artifacts so that expression may be compared within or between samples. As discussed in (49), many systematic sources of variation affect a gene’s observed expression level and should ideally be adjusted for including capture efficiency, amplification biases, differences in total RNA content, and sequencing depth of the spot (or region) at which expression is measured. It is difficult to estimate many of these factors in practice, however, and consequently most often normalization amounts to adjusting for differences in sequencing depth. Some of the most popular approaches for scRNA-seq - logged transcripts per million (log(TPM+1)) or scran - have become the most widely used for ST experiments although it is becoming clear that new methods would prove useful, especially in ST experiments where cell density is highly variable (50). In these experiments, normalization methods that account for the effect of cell density on total counts per spot are required.

Following normalization, clustering is often done to identify cell subtypes followed by characterization either by identifying marker genes that are highly expressed in the cluster, or those highly expressed relative to other clusters via differential expression (DE) analysis. While the H&E stain (or related image) provides much insight into cell subtypes, molecular determination of subtypes can lead to refined characterizations.

Methods for clustering and identification of DE genes that were widely used for scRNA-seq continue to be used in ST data analysis. These include k-means and graph-based methods such as Louvain, and Leiden for clustering; and the Wilcoxon rank-sum test and DESeq for identification of DE genes. These latter DE methods do not appropriately accommodate replication at the cell and sample level, and, because of this, pseudo-bulk approaches have been recommended (51). Unfortunately, the recommendation was largely based on a biased assessment and pseudo-bulk approaches are proving to be critically underpowered (52); mixed effects models provide a good balance that accommodates sample-to-sample variability while preserving information about variability cell-to-cell (52). In addition to approaches well-established in scRNA-seq analysis that are being applied in the ST domain, novel methods are beginning to emerge that leverage correlation inherent in spatial data specifically. BayesSpace, for example, leverages information from spatial neighborhoods to improve cluster identification; it also allows for the identification and characterization of cell types at sub-spot resolution. Other methods such as SpaGCN and MUSE incorporate histology information to identify clusters. Similarly, many methods including Trendsceek, scHOT, SpatialDE, SPARK, and SpatialDE2 have been developed to identify genes with average expression that varies spatially (53–56); SpatialCorr can be used to identify spatial changes in the correlation between pairs (or groups) of genes (57). While these approaches have proven invaluable for characterizing gene expression across a tissue, the technology is limited to expression derived from multiple cells. Consequently, deconvolution and/or mapping methods that integrate ST with single-cell data are required to obtain a more detailed characterization of the cell-type architecture underlying tissue regions, and to infer cellular networks (58). Deconvolution methods that combine ST with scRNA-seq data can be used to estimate the proportions of specific cell types present at each spot (59–65). While useful, most of these methods assume that the spatial and single-cell datasets have congruent cell types, and biases in inferred cell fractions are introduced when this is not the case (e.g. when cell types are present in the ST data, but not present in the scRNA-seq reference dataset). Recent methods such as spSeudoMap are being developed specifically to mitigate this type of bias (60). Since deconvolution methods provide estimates of cell type proportions, but not expression estimates for individual cells, they cannot be used to discover spatially determined cell states and/or to infer cell-cell interactions. Recent methods such as CytoSPACE (66), COMMOT (67), and SpaTalk (68) have been developed toward this end.

In summary, the methods most used for analysis of ST data were initially developed for bulk or scRNA-seq. A typical workflow for Visium data uses SpaceRanger for alignment using the default method STAR; data are then often imported into Seurat for normalization, clustering, and DE analysis via the default methods scran, k-means on UMAP principal components, and the Wilcoxon rank-sum test, respectively. GeoMx provides a similar pipeline with STAR for alignment followed by estimating gene expression levels, filtering, normalization, and further analysis. As discussed above, many methods have been developed specifically for ST data that improve upon the basic methods implemented in SpaceRanger and GeoMxTools. Most were developed for Visium data with applications to GeoMx data possible, with the caveat that some methods require contiguous spots which are not available in GeoMx. In addition, there may be technical artifacts in GeoMx data that are not accommodated by common statistical assumptions and new methods are required (69).

## Glioblastoma tumor heterogeneity and microenvironment

Intratumoral heterogeneity of the GBM tumor microenvironment (TME) is the difference in genetic, molecular, and physical structures of the tumor cells, and their native cell counterparts, all present within the native tumor microenvironment (70, 71). Because of the multitude of complex cell types present within the normal brain, intratumoral heterogeneity of GBM is vast and complicated by the interaction between the tumor cells and the surrounding tissue. These differences in genetic makeup and unique microenvironmental interaction are thought to play a role in treatment resistance and disease recurrence (72, 73). With the evolution of the molecular technologies and computational advances of more robust sequencing methods it has been observed that GBM possesses remarkable heterogeneity both within single tumors as well as across individual patients. Increase in the heterogeneity of a particular GBM has been shown retroactively to impact survival (70). The importance of tumoral heterogeneity on prognosis and development of therapeutics for pathologies such as cancer has long been evident. To systematically categorize this heterogeneity across a wide range of cancer The Cancer Genome Atlas (TCGA) project was started. TCGA compiles oncogenomic, methylomic, transcriptomic and proteomic data from tumor samples that were annotated with clinical (and in some cases MRI) data (74). This bulk database serves as a foundational catalog of genomic abnormalities that drive tumorigenesis, by categorizing the genomic changes of a large cohort of cancer samples (38). Subgroup analysis using specifically GBM tumors revealed associated mutations such as PIK3R1, NF1, and ERBB2. This genomic information enabled GBM to be sub-typed and further classified at the level of RNA expression into proneural, neural, and mesenchymal subtypes (38). The end goal of this gene-based classification is to understand if it’s possible to target more focused and personalized inhibitors to the specific subtypes of GBM rather than one-sized fits all chemotherapy and radiotherapy approaches.

As sequencing technology advanced and single cell RNA seq (scRNAseq) was developed further interrogation of GBM tumor samples uncovered more complex “hybrid” states at the single cell level. In these states bulk tumors were found to consist of heterogeneous mixtures of individual cells corresponding to different GBM subtypes, rather than every cell within a tumor being a consistent subtype (35). This finding reflects aberrant developmental programs or interconversion between phenotypic states, leading to crossing over of the various subtypes with regards to their RNA profile (35). Hybrid states were most notably detected among classical and proneural subtypes (progenitor states) or the mesenchymal and neural substrates (differentiated states). Later research defined developmental arc’s between proneural/classical subtypes and the mesenchymal subtype, as well as a propensity towards a shift to the mesenchymal subtype after standard of care therapy (39, 75). This granular information obtained from scRNAseq, which may be critical to developing successful therapies in the future, was covered up in bulk RNAseq due to average based cutoffs biasing for only large effect sizes.

Within the GBM TME there are also a myriad of complex cell types that makeup the surrounding normal brain tissue as well as the patrolling and invading lymphocytes attempting to respond to tumor growth. These cell types include but are not limited to microglia, neurons, macrophages, T-cells, MDSC’s, astrocytes, and oligodendrocytes (24, 33, 41, 72, 73, 76). As the composition of the TME has been more fully elucidated with research studies looking into the functional consequences of interaction with these surrounding cells have begun to illuminate a dynamic cellular crosstalk between GBM itself and its TME. Studies examining GBM and its complex TME have uncovered critical insights including wide immunosuppression (29, 77–80), integration with neuronal firing (81–84), hijacking of angiogenesis (85–89), changes in cellular metabolism (29, 90, 91), and disruption of normal ph. and oxygen levels (29, 92).

Although scRNAseq is a clear advancement over bulk RNAseq in furthering our understanding of GBM heterogeneity and has significantly added to the identification of potential therapeutic molecular targets, it is still limited in its ability to capture cellular behavior in its native ecosystem. Furthermore, FISH can provide true spatial gene expression information but due to the limitations of multiplexing is only available for a small number of targets. The culmination of all this research gives a glimpse of how complex both GBM and its TME are, however, until the advent spatial transcriptomics a final piece of the puzzle remained missing.

## Spatial transcriptomics in glioblastoma

Initiatives such as Ivy Gap have made significant contributions to understanding glioblastoma and its complex microenvironment, however, its limited sample size along with the need for sample dissociation prior to sequencing are critical limitations. These limitations make clear the need for a sequencing method that can take spatial location into account and allowing for a more complete picture of GBM TME and its interaction with the surrounding tissue microenvironment.

Recent work involving both single cell and spatial methodologies is just beginning to highlight novel and promising discoveries (**Table 1**) that may potentially lead to the next therapeutic breakthrough. The most complex study done to date integrating spatial transcriptomics with metabolomics and proteomics (through MALDI and Mass Cytometry) was conducted by Ravi, Will, and Kueckelhaus et al (76). The authors discovered 5 unique and spatially distinct transcriptomics profiles among their 28 sample GBM cohort termed radial glia, reactive immune, neuronal development, spatial OPC and reactive hypoxia. Validation of both the bulk sequencing original molecular subtypes (38) and further single cell transcriptomic profiles (35, 75) was also conducted but not noted to have significant spatial enrichment within a specific tumor location among the analyzed samples. The most interesting observation from that study is the metabolic shift between glycolysis and the pentose phosphate pathway within the spatially localized necrotic core of tumors (reactive hypoxia compartment) leading to an accumulation of copy number variation mutations in the surrounding cells due to environmental pressures of low oxygen driving DNA mutations. The authors hypothesize that these highly mutated cells may be the source of some of the remarkable plasticity under therapeutic pressure exhibited by GBM as they migrate between the hypoxic/necrotic core and the leading edge of the tumor (76). A critical weakness of the study, noted by the authors within the manuscript, is the bias towards regions with a dense population of tumor samples rather than more sparse regions to allow for the integration of scRNA sequencing and spatial sequencing.

**Table 1:** All published primary spatial transcriptomics studies in GBM.

Title	Authors	Publication Date/Journal	Spatial Technology	Major finding	Samples used	Number of samples
***Spatially resolved multi-omics deciphers bidirectional tumor-host interdependence in glioblastoma***	Vidhya M. Ravi, Paulina Will, Jan Kueckelhaus, Na Sun, Kevin Joseph, Henrike Salie, Lea Vollmer, Ugne Kuliesiute, Jasmin von Ehr, Jasim K. Benotmane, Nicolas Neidert, Marie Follo, Florian Scherer, Jonathan M. Goeldner, Simon P. Behringer, Pamela Franco, Mohammed Khiat, Junyi Zhang, Ulrich G. Hofmann, Christian Fung, Franz L. Ricklefs, Katrin Lamszus, Melanie Boerries, Manching Ku, Jurgen Beck,Roman Sankowski,Marius Schwabenland,Marco Prinz, Ulrich Schuller, Saskia Killmer, Bertram Bengsch, Axel K. Walch, Daniel Delev, Oliver Schnell, Dieter Henrick, Heiland	June 13, 2022, Cancer Cell	Visium 10X	Found the spectrum of regional transcriptional programs of GBM, mapped their microenvironmental landscape including metabolic and tumor-host cellular interactions. Uncovered insights into the bi and unidirectional interactions between the microenvironment and the spatial-temporal alterations in transcriptional heterogeneity of GBM. Found a strong association between inflammatory response and the age of the donor, suggesting that aging may have an impact on tumor differentiation. Was concluded that tailored therapeutic approaches are required.	Patient tissue specimens	28
***T-cell dysfunction in the glioblastoma microenvironment is mediated by myeloid cells releasing IL10***	Vidhya M. Ravi, Nicolas Neidert, Paulina Will, Kevin Joseph, Julian P. Maier, Jan Kückelhaus, Lea Vollmer, Jonathan M. Goeldner, Simon P. Behringer, Florian Scherer, Melanie Boerries, Marie Follo, Tobias Weiss, Daniel Delev, Julius Kernbach, Pamela Franco, Nils Schallner, Christine Dierks, Maria Stella Carro, Ulrich G. Hofmann, Christian Fung, Roman Sankowski, Marco Prinz, Jürgen Beck, Henrike Salie, Bertram Bengsch, Oliver Schnell, Dieter Henrik Heiland	February 17, 2022, Nature Communications	Visium 10X	Combine single-cell RNA sequencing along with spatially resolved transcriptomic sequencing to gain spatial insights into the complex crosstalk, cellular states, and cellular plasticity leading to the immunosuppressive environment found in GBM. Spatial transcriptomics was used to identify the spatial overlap of cells; was observed that the HMOX1+ myeloid cells were spatially correlated with T cell exhaustion and the mesenchymal state of glioblastoma. Also confirmed the presence of HMOX1+ myeloid cells to result in the reduction of the effector t cell population.	Patient tissue specimens	8
***Spatial transcriptomics reveals niche-specific enrichment and vulnerabilities of radial glial stem-like cells in malignant gliomas***	Yanming Ren, Zongyao Huang, Lingling Zhou, Peng Xiao, Junwei Song, Ping He, Chuanxing Xie, Ran Zhou, Menghan Li, Xiangqun Dong, Qing Mao, Chao You, Jianguo Xu, Yanhui Liu, Zhigang Lan, Tiejun Zhang, Qi Gan, Yuan Yang, Tengyun Chen, Bowen Huang, Xiang Yang, Anqi Xiao, Yun Ou, Zhengzheng Su, Lu Chen, Yan Zhang, Yan Ju, Yuekang Zhang, Yuan Wang	February 23, 2023, Nature Communications	Visium 10X	Integrated short and long read spatial transcriptomic datasets to provide a comprehensive spatial profiling of DMG and GBM. Revealed niche-specific glioma ecosystems and regulatory programs. Identified four gene expression modules that are conserved across tumor samples, and showed the similarities that DMB and GBM share, despite its unique driver mutations and age of onset.	Patient tissue specimens	10
***Highly Multiplexed Spatially Resolved Proteomic and Transcriptional Profiling of the Glioblastoma Microenvironment Using Archived Formalin-Fixed Paraffin-Embedded Specimens***	Youngmi Kim, Patrick Danaher, Patrick J Cimino, Kyle Hurth, Sarah Warren, John Gold, Joseph M Beechem, Gabriel Zada, Troy A McEachron	January 1, 2023, Modern Pathology	NanoString GeoMx	Spatial tech allowed for highly multiplexed digital antibody-based protein profiling on serial sections for an integrated multianalyte approach. Demonstrated applicability of an integrated multiparametric approach to characterize the tumor microenvironment of archived formalin-fixed tissues.	Patient tissue specimens	3
***Advanced Molecular Characterization Using Digital Spatial Profiling Technology on Immunooncology Targets in Methylated Compared with Unmethylated IDH-Wildtype Glioblastoma***	H Barber, A Tofias, B Lander, A Daniels, J Gong, Y Ren, X Ren, Y Liang, P White, K M Kurian	February 24 2021, Journal of Oncology	NanoString GeoMx	There was a statistical increase in specific ROIs (CD4, CD14, CD68, CD8A, B7-H3, PDL-1, CD19, FOXP3, CD44, and STAT3) protein expression in methylated versus unmethylated GBM tumor core.	Patient tissue specimens	19

Ravi et al. conducted a similar study using 12 patient tumor samples but this time focusing less on the tumor spatial structure and more on the structure of the TME, and specifically the tumor surrounding T-cells (93). They define a specific subset of myeloid cells that are HMOX1+ tend to localize spatially to mesenchymal like tumor regions and drive T-cell exhaustion. The release of IL-10 is said to be the main driver of this T-cell immunosuppression which can be rescued through inhibition of the JAK-STAT pathway. Barber et al. also conducted a spatial transcriptomics study comparing core and periphery of GBM IDH-WT samples that were either MGMT methylated or unmethylated (94). They found a significant increase in proteins such as CD4, CD14, CD68, CD8A, B7-H3, PDL-1, CD19, FOXP3, CD44, and STAT3 was associated within the cores of methylated tumors but not unmethylated tumors. Furthermore, this difference was not seen when peripheral edges were compared, possibly indicating that cells with the most diversity resided away from the rapidly advancing peripheral tumor edge (94).

Ren and colleagues examined both diffuse midline glioma as well as GBM using spatial transcriptomics and note that both tumor types display niche specific microenvironments and transcriptional programs (95). Specifically, tumor cores were enriched for oligodendrocyte precursor like cells while radial glia like stem cells are enriched within the invasive niche. This invasive niche was also found to be the most neuron rich spatial environment. Along with these spatial niches there are corresponding regulatory programs and drivers such as FAM20C that drive the invasive growth of the radial glia stem like cells along the proliferative edge. Finally, Kim et al. corroborated these niche specific transcriptional programs as well as differing response to hypoxia within tumor samples (96). It was observed that hypoxia specific response genes were found mainly in VEGF low spatial ROI’s although the authors note patient to patient variability in this response. Furthermore, neuropathologic findings was found to positively correlate with computationally assigned scores in both microvascular proliferation and overall neoplastic scores indicating that the computational deconvolution used throughout this study was indeed real world validated (96).

Taken together, these early studies conducted thus far using spatial transcriptomics on GBM and other high grade brain tumors definitively show that there are region specific and distinct gene expression programs throughout the tumors. These programs display similar amounts of intertumoral heterogeneity as was hypothesized when bulk and single cell sequencing studies were conducted in GBM, however, in some of the larger sample studies, patient specific variations can be observed such as the hypoxic niche and proliferative edge programs (76). Critically, the literature is still inconclusive between the true functions of these transcriptional and spatial niches as *in vivo* and *in vitro* controlled experimentation is needed.

## Spatial transcriptomics and its role in future of personalized medicine

The most challenging aspect of any new research technology is its eventual integration into the clinic to try to better patient outcomes. GBM represents a disorder desperately in need of new therapeutic developments ranging from prognostic biomarker discovery to novel interventions aimed at prolonging survival. To successfully translate new discoveries into clinic the barriers of efficacy and safety, which are much higher than the common basic science barrier of statistical significance, must be surpassed. This translational step is where many of the current bioinformatic advances in GBM have fallen short. For example, studies have demonstrated repeatedly that specific molecular subtypes exist within patients (35, 38, 75), and that tumor heterogeneity is pervasive, however, no clinical benefit or defined molecular target has yet to emerge. A patient presenting to the clinic with proneural GBM and a highly heterogeneous tumor (as defined by NGS) is treated exactly the same as a patient presenting with low tumor heterogeneity and a mesenchymal subtype. To date the only significant prognostic markers that exist in the GBM space are IDH mutation status and MGMT methylation status. It’s possible that because GBM is such a spatially organized tumor itself, and it resides in such a spatially complex organ system, that gene expression values must be analyzed within the context of spatial information to be clinically relevant.

An avenue of clinical research that could directly benefit from spatial transcriptomics is the integration between GBM and cells that make up its TME. A novel player in the GBM/TME interactions is surrounding neurons. New evidence supports a key role played by neuron cells in glioma’s TME through the existence of neural-glioma networks (81–83). These involve both bona fide neuron-glioma synapses and non-synaptic interconnections forming an electrically coupled network involving multiple feedback loops through which neurons regulate glioma growth. The identification of the classic synaptic AMPA receptor mediated pathways as a key element in the neuron-glioma networks underpins the finding that AMPAR-blocking drugs (such as perampanel) can successfully inhibit glioma proliferation in murine models, demonstrating the potential for agents addressing TME-glioma crosstalk as a therapeutic strategy. Studies have also supported important roles played by other TME cells such as tumor-infiltrating lymphoid and myeloid immune cells (93, 97–99). These immune infiltrates in GBM are largely immunosuppressive, in part due to T-cell dysfunction/exhaustion, which can be partly explained by crosstalk between myeloid and lymphoid cells (100, 101). This immunosuppression has been clinically manifested in the failure of various types of immunotherapy treatments trialed in GBM. Spatial transcriptomics may illuminate novel connections or gene expression patterns within niche areas that promote neuronal/GBM interactions or conversely demonstrate that some areas of the tumor such as the hypoxic core contain the most immunosuppressive signatures.

Another avenue for spatial transcriptomics to make clinical inroads is in discovery of novel biomarkers of disease progression. Biomarkers of response to standard of care in gliomas remain extremely limited, with MGMT promoter methylation and IDH mutation status as the only predictive biomarkers currently in clinical use in malignant gliomas (102). This dearth of biomarkers can be largely explained by the limited understanding of glioma’s unique TME. Spatial transcriptomics holds great promise in enabling a better understanding of how TME influences tumor evolution and response to therapy. Examples of the TME’s role in uncovering patient response to therapy among other cancers include the role of tumor-infiltrating lymphocytes (TILs) in predicting response of HER-2 negative breast cancer to paclitaxel-based regimens (103). Efforts at characterizing other tumor’s TME have resulted in novel classifying schemes of treatment response, with examples in hypopharyngeal carcinoma suggesting its role in distinguishing responders from non-responders (104). This raises the potential of similar advances in GBM as more evidence emerges of GBM’s specific TME biology.

The effects of different therapeutics on GBM and its TME can also be more fully taken into consideration using spatial transcriptomics. The gold standard chemotherapy for GBM is temozolomide (TMZ), however, the impact of temozolomide in the TME modulation remains unclear, with some reports suggesting its potential use as an enhancer of immune activation in regimens combining TMZ with immunotherapy (105), whilst others have suggested its contribution to the TME’s immunosuppressive behavior through Treg enrichment, partially reversible through IL-2 (27, 106). Radiation which is also part of the standard of care in GBM treatment is also not fully explored spatially with evidence suggesting there is existence of potential targets to radiosensitize gliomas, including TGF-B (107). However, recent evidence has also supported the potential role of radiation in transforming glioma’s TME into a tumor-permissive environment (107, 108) with astrocyte-derived transglutaminase 2 (TGM2) secreted by irradiated astrocytes contributing to tumor stemness and radioresistance.

Immunotherapy represents another example of the importance of the TME and tumor-TME interactions. In GBM, multiple approaches have been attempted and continue to be proposed, including CAR-Ts (85, 109–111), dendritic cell vaccines (112–114), immune checkpoint blockade (79, 86, 115), oncolytic virus (116–119) and others. While human clinical trials have been mostly negative so far, emerging evidence has supported the role of TME in identifying and in conditioning response to immunotherapy. Spatially categorized tumor explants and spatial-omics have begun enabled a more comprehensive 3-Dimensional understanding of immune cells distribution in gliomas, with implications in predicting response to immunotherapy (120). As spatial transcriptomics develops further this response prediction could be further integrated with other data such as tumor mutational burden and neoantigen load to create a more comprehensive prediction of likely response to immunotherapy.

**Figure 3:**
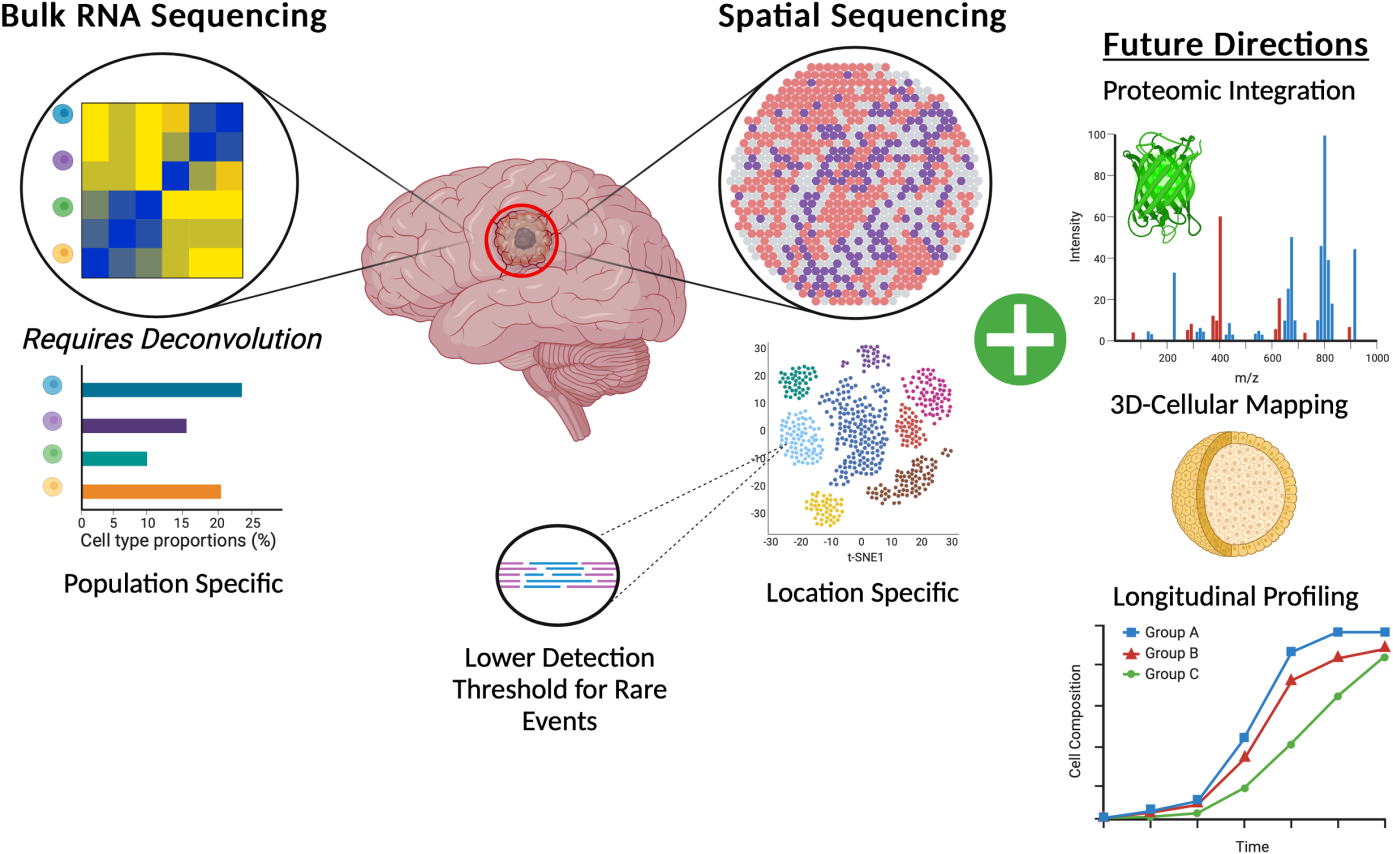
Graphic representation of the benefits of spatial transcriptomics for GBM brain tumors when compared to canonical bulk RNA-Sequencing and also highlighting potential future directions of spatial transcriptomics.

Across the clinical landscape of GBM there are a wealth of opportunities to leverage spatial transcriptomic data to create more specific and accurate predictors of response. A change in the design of upcoming clinical trials can also consider the spatial transcriptomic landscape of tumors from patients on the study offering personalized medicine specific therapies based on the profile of an individual patient’s tumor. These more spatially centric basic science studies and clinical trials can further improve the discovery of novel effective drugs and patient responders (**Figure 3**).

## Conclusions and future directions

Advances in spatial transcriptomics may hold the key to unraveling the highly nuanced biology behind all manner of complex processes from development to disease. As further technological advances are made within the field, integration of proteomic data with spatial location seems to be a crucial next point of exploration (96). Further integration of spatially resolvable data with techniques such as metabolomics or sequencing at the single cell level will also no doubt advance our collective knowledge. The authors of many of the early spatial transcriptomics papers note that this technology is not in direct competition with assays such as proteomics or scRNAseq, rather that they should be used in concert to draw more data dense and meaningful conclusions. Indeed, integration of these technologies with other technologies such as proteomics, metabolomics, tissue multiplexing, and the development of higher single cellular resolution is already underway (**Table 2**). The transition from largely two-dimensional thin tissue slides into full three-dimensional organoids will also push the field of spatial transcriptomics forward. Researchers have begun to push this boundary with 3D metabolic profiling in tonsil tissue with Mass Spectrometry ion beams measuring and stacking 5 nanometer sections across a total of 150 sections of human tonsil (121). As more complex integration of data is performed, more nuanced bioinformatics and statistical corrections also need to be put in place to handle the resulting highly dimensional and variable datasets. Although the advances possible with this technology are exciting and within reach, care needs to be taken to accurately check and QC the resulting data. More accepted and universal bioinformatics pipelines that are maintained and standardized (as is the case for WES and RNA sequencing) will also be critical in ensuring the accuracy and reproducibility of results from these studies.

**Table 2:** Current spatial transcriptomics services and platforms. (*Offered as of the time of publication of this review.)*.

Platform	Company	Spatial Technology	Cellular Resolution	Uses/Highlights
**GEOMX**	Nanostring	Whole Transcriptome with possible protein targeting, ROI selection needed	10um	Cellular barcodes can be quickly counted on Ncounter machines or NGS sequenced. Many off the shelf targeting panels available.
**COSMX**	Nanostring	Whole Transcriptome with possible protein targeting	Single cell or subcellular	Currently in development/limited release. Similar to GEOMX but greater resolution.
**Visium**	10x Genomics	Full gene expression profiling using NGS no ROI selection needed	1-10 cells per spot	Can be integrated with protein detecting using IHC. Can detect transcripts across the entire tissue section.
**Xenium**	10x Genomics	Detection of 100’s to 1000’s of RNA targets as well as multiplexed protein	Single cell or subcellular	Can be expression tuned as well as detect highly degraded transcripts.
**Cell DIVE**	Leica	Multiplex imaging	Whole tissue imaging down to single cell level	Possible linking of multiplex imagers through software to enable higher throughput research.
**Lunaphore-Comet**	Lunaphore	Multiplexed Imaging	Whole tissue imaging	Easy project design with use of off the shelf antibodies.
**MERSCOPE**	Vizgen	Multiplexed Imaging (MERFISH)	In-Situ single cell spatial genomics	Ability to profile large tissue sections down to cellular/subcellular levels with high sensitivities and multiplexing possibilities.
**Chip Cytometery**	Canopy Biosciences	Multiplex Imaging	Whole tissue imaging	Serial multiplexing allows for use of many chosen markers as well as reintegration of sample at a later time.

Within the landscape of disease, the greatest challenge will be drawing not only biologically meaningful but clinically meaningful conclusions and data from these experiments. Current research further characterizing the subtypes of GBM have yielded little clinical results thus far, perhaps the combination of spatial data from both the TME and native tissue microenvironment can enable discovery of targeted treatments. New directions for spatial sequencing experiments in GBM can begin to explore more in-depth tumor-neuronal interactions which have already been documented in the literature (81–83). Direct contact with tumor trafficked immune cells or surrounding astrocytes or microglia can also be more thoroughly investigated as it’s been hypothesized that GBM can assert its effects both locally through contact as well as more globally through manipulation of cytokine and chemokine secretion (122–125). Furthermore, as spatial technology continues to approve and resolution approaches the single cell level locations along the edges of tumor and normal brain containing more sparse cell populations, rather than densely populated tumor areas, can begin to be more closely examined. Study of these regions may prove vital as they are on the edge of a rapidly advancing tumor and may display a preconditioned phenotype necessary for eventual tumor growth.

Finally, the last critical dimension of sequencing that has eluded the biological and bioinformatics community is time. Over time spatial architecture changes and interactions between cells are formed and broken, with the power to now see these interactions in their native spatial landscape revolutionary discoveries stand to be made both in normal and disease models. This type of technological advance would be well suited for fields such as embryology or brain development where expression changes over time drive vast cellular architectural changes. These experiments could also be utilized for *in vitro* organoid modeling of things such as vascular development, or the growth of tumors from an early seeding stage. Although this could be somewhat crudely done currently with just serial spatially profiled experiments, technological advances in microscopy and sequencing that would allow for continuous profiling of a single specimen could be revolutionary. Additionally, with serial spatial experiments, as well as the development of technologies that can label cell interactions such as RABID seq (126), authentic cellular interaction can be visualized to understand normal developmental trajectories as well as abnormal disease progression.

To truly make meaningful gains in patient survival in a disease as devastating as GBM researchers need to make use of all the tools at their disposal. Spatial transcriptomics represents a worthwhile tool added to the investigator arsenal, however, our tools are only as good as the questions we use them on. As our methods evolve so too must our hypotheses in order to fully realize our ultimate goal of ending GBM.

## Author contributions

JS: Writing – original draft, Writing – review & editing, Conceptualization, Project administration, Visualization. LC: Writing – original draft. AG: Writing – original draft. DG: Writing – original draft. SP: Visualization, Writing – review & editing. AQ-H: Supervision, Writing – review & editing. CK: Supervision, Writing – review & editing. MD: Supervision, Writing – original draft, Writing – review & editing.
